# The Potential of Circulating Cell-Free DNA Methylation as an Epilepsy Biomarker

**DOI:** 10.3389/fncel.2022.852151

**Published:** 2022-03-24

**Authors:** Ricardo Martins-Ferreira, Bárbara Guerra Leal, Paulo Pinho Costa

**Affiliations:** ^1^Epigenetics and Immune Disease Group, Josep Carreras Research Institute (IJC), Barcelona, Spain; ^2^Immunogenetics Lab, Molecular Pathology and Immunology, Instituto de Ciências Biomédicas Abel Salazar—Universidade do Porto (ICBAS-UPorto), Porto, Portugal; ^3^Autoimmunity and Neuroscience Group, Unit for Multidisciplinary Research in Biomedicine (UMIB), ICBAS-UPorto, Porto, Portugal; ^4^Laboratory for Integrative and Translational Research in Population Health (ITR), Porto, Portugal; ^5^Instituto Nacional de Saúde Dr. Ricardo Jorge, Department of Human Genetics, Porto, Portugal

**Keywords:** cell-free DNA, DNA methylation, epilepsy, epileptogenesis, MTLE-HS, biomarker

## Abstract

Circulating cell-free DNA (cfDNA) are highly degraded DNA fragments shed into the bloodstream. Apoptosis is likely to be the main source of cfDNA due to the matching sizes of cfDNA and apoptotic DNA cleavage fragments. The study of cfDNA in liquid biopsies has served clinical research greatly. Genetic analysis of these circulating fragments has been used in non-invasive prenatal testing, detection of graft rejection in organ transplants, and cancer detection and monitoring. cfDNA sequencing is, however, of limited value in settings in which genetic association is not well-established, such as most neurodegenerative diseases.Recent studies have taken advantage of the cell-type specificity of DNA methylation to determine the tissue of origin, thus detecting ongoing cell death taking place in specific body compartments. Such an approach is yet to be developed in the context of epilepsy research. In this article, we review the different approaches that have been used to monitor cell-type specific death through DNA methylation analysis, and recent data detecting neuronal death in neuropathological settings. We focus on the potential relevance of these tools in focal epilepsies, like Mesial Temporal Lobe Epilepsy with Hippocampal Sclerosis (MTLE-HS), characterized by severe neuronal loss. We speculate on the potential relevance of cfDNA methylation screening for the detection of neuronal cell death in individuals with high risk of epileptogenesis that would benefit from early diagnosis and consequent early treatment.

## Introduction

The search for effective biomarkers is one of the most challenging tasks in epilepsy research. Epileptogenesis is now considered a progressive process, with seizure activity contributing to extensive damage and seizure recurrence (Pitkänen et al., 2015). Epilepsy diagnosis must thus be viewed as a race against the clock, aiming for the detection, as early as possible, of individuals with a high risk of epilepsy development, that could be candidates for preventive pharmacological intervention. The search for novel viable biomarkers is considered crucial to improve the current hurdles to early diagnosis and prognostic monitoring in epileptic patients. A major issue continues to be the high refractoriness rates (estimated at 30%) across different types of epilepsy (Pitkänen et al., 2016). Epilepsy treatment is based on the control of ictogenesis. Current anti-seizure drugs target seizure control and are not directed to the whole epileptogenic landscape. Additionally, such biomarkers would facilitate economically feasible clinical trials for the development of new and more effective anti-epileptic therapies by allowing a more precise categorization of individuals with high probability of developing epilepsy (Engel and Pitkänen, 2020).

Nucleic acid-based disease biomarkers have represented one of the most exciting developments in recent years, with non-coding RNAs, specifically microRNAs, gaining center stage (Henshall et al., 2016). In this review, we consider the potential of the still largely unexplored circulating cell-free (cf) DNA. We will focus on cfDNA methylation and its ability to predict cell and tissue of origin in epilepsies with a histopathological phenotype of neuronal damage and death.

## Biogenesis and The Apoptotic Cascade

The first description of cfDNA in human plasma dates to 1948 (Mandel and Metais, 1948). Nowadays, it is broadly described as highly degraded DNA fragments circulating freely in the peripheral blood. However, the full spectrum of processes responsible for cfDNA production and release remains elusive. There is a perception that cfDNA may be originated from countless biological features, both physiological and pathological, such as inflammation, aging, exercise, or cancer. The main mechanisms of cfDNA release are considered to be active release or cellular breakdown (Aucamp et al., 2018). The overall conception, on which most of the studies in this field base their hypotheses, is that cfDNA originates predominantly from apoptosis. This relies on the matching sizes between cfDNA and DNA fragments resultant from the apoptotic cascade. Caspase-activated DNase is one of the main effectors of DNA fragmentation during apoptosis. It is a double-stranded endonuclease which lacks exonuclease activity, thus being only capable of fragmenting DNA in inter-nucleosomal linker regions (Heitzer et al., 2020). Human circulating cfDNA presents a predominant and consistent fragment length of approximately 167 bp (Snyder et al., 2016), which corresponds to the length of DNA wrapped around a nucleosome (~147 bp) plus linker fragments (Heitzer et al., 2020).

## Genetic Sequencing and Liquid Biopsies

In practice, the study of cfDNA has consisted predominantly of its genetic characterization. Sequencing of cfDNA is nowadays routinely used in some clinic settings. Screening of fetal DNA circulating in the mother’s bloodstream, non-invasive prenatal testing, is used to detect chromosomal abnormalities (Fan et al., 2012). Transplant graft rejection is also monitored by the detection of donor-derived cfDNA sequences (De Vlaminck et al., 2014). Throughout the last two decades, cfDNA sequencing has been increasingly applied to cancer detection (Chabon et al., 2020) and also in post-therapy prognosis (Nabet et al., 2020; Powles et al., 2021). These liquid biopsies provide for non-invasive detection of tumor DNA in biological fluids, also denominated as circulating tumor DNA.

The relevance and potential of cfDNA does not end in its genetics, though. The utility of nucleotide sequencing is narrow in non-mutation rich pathologies, meaning conditions in which a clear association with a mutated genetic profile is not observed. These include a considerable number of neurodegenerative diseases such as, in the interest of this review, different forms of epilepsy.

## Deconvoluting Cell-Of-Origin Through DNA Methylation

**Figure 1:**
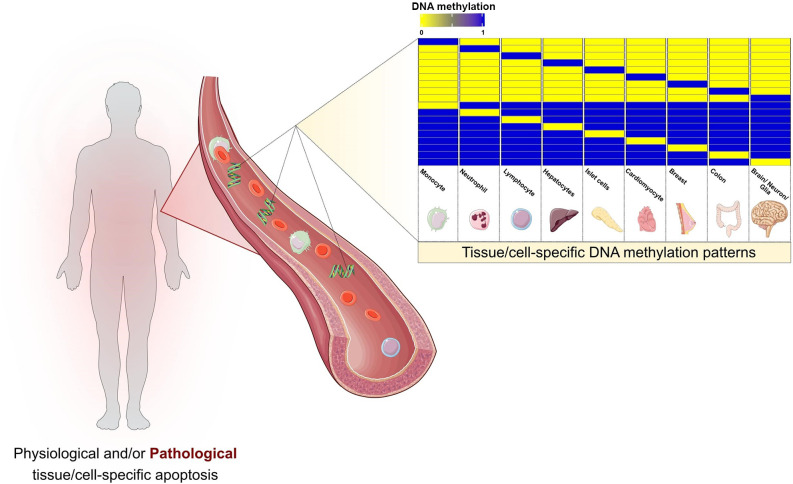
Cell damage and apoptotic events during both physiological and pathological conditions are thought to release cell-free DNA (cfDNA) into the bloodstream. The pool of cfDNA in circulation present specific DNA methylation patterns that would allow the detection and localization of cell death, opening the doors for a wide spectrum of prospective clinical applications. The selected cell and tissue types are intended to be representative. Hematopoietic cells, the main contributors to cfDNA release, are depicted by monocytes, neutrophils, and lymphocytes. Hepatocytes, pancreatic islet cells, cardiomyocytes, breast, and colon are some examples of tissues with previous evidence on the estimation of cell/tissue of origin based on cfDNA methylation. Brain, neurons, and glia represent the potential of applying such pipelines in neurodegenerative diseases.

The strong association between cfDNA and apoptosis suggests the occurrence of cell death somewhere in the organism. However, genetic screening of the circulating DNA is unable to specifically identify tissue or cell of origin. In this context, epigenetics and, specifically, DNA methylation have entered and significantly upgraded cfDNA research. DNA methylation consists of the covalent addition of a methyl group to cytosine residues in the genomic sequence. It occurs predominantly in CpG dinucleotides and is associated with transcriptional regulation. Certain patterns of DNA methylation are highly tissue and cell-specific and conserved in both physiologic and pathologic conditions (Dor and Cedar, 2018; Figure 1).

Alterations of DNA methylation in cfDNA may result from alterations in specific cells or tissue between the pathologic and disease-free settings. Additionally, it can also originate from changes in the composition of cfDNA due to differential cell death rates (Figure 1). The analysis of the cfDNA methylome has been conducted in two main lines of thought. The first opts for directly calculating differential methylation patterns, such as differentially methylated positions (DMPs) and differentially methylated regions (DMRs) between different conditions (Hatt et al., 2015; Jensen et al., 2015; Legendre et al., 2015; Xu et al., 2017; Gallardo-Gómez et al., 2018; Gordevičius et al., 2018). However, the direct usage of cfDNA profiles as predictors may be associated with an increased signal-to-noise ratio, since DMPs and even DMRs can be non-specifical and more difficult to interpret biologically. Cell or tissue-of-origin estimation may be considered as a reduction step, decreasing background noise, and increasing prediction accuracy (Feng et al., 2019). Prediction of cfDNA origin can be approached through two different strategies. The first consists of targeted DNA methylation analysis, that is predicting cell-of-origin by searching for previously identified specific DNA methylation alterations characteristic of a cell type (DMPs and DMRs). This approach has been applied throughout a wide spectrum of settings, including the detection of cfDNA derived from pancreatic β-cell in Type 1 diabetic patients (Olsen et al., 2016a), cardiomyocytes in cardiac infarction and sepsis patients (Zemmour et al., 2018) and hepatocytes in liver transplant recipients, individuals who underwent hepatectomy and sepsis patients (Lehmann-Werman et al., 2018). The second approach consists of genome-wide deconvolution tools that have been developed to simultaneously estimate the percentages of the contribution of multiple tissue and cell types to circulating cfDNA. Deconvolution algorithms have been predominantly used in cancer DNA methylation research (Sun et al., 2015; Guo et al., 2017; Kang et al., 2017; Li et al., 2018). A study by Moss et al. (2018) developed a comprehensive reference-based deconvolution tool accounting for a total of 25 cell/tissue types obtained from 450k and EPIC arrays of primary tissue sources. They validated the previous conception that cfDNA in plasma of healthy individuals as originating predominantly from hematopoietic cells. Furthermore, the authors demonstrated promising results in pathological contexts, by detecting increased plasma cfDNA of pancreatic origin in islet transplant recipients, significant correlation of hepatocyte-derived cfDNA and clinical liver damage markers, and detection of tissue-specific cfDNA in three cancer settings (colon, lung, and breast). Interestingly, cfDNA estimated origin showed good prognostic ability in prostate cancer patients after treatment, and also showed promise in the prediction of tissue of origin in cancer patients with no clear primary (Cancer of Unknown Primary; Moss et al., 2018). An additional interesting aspect of this study is the fact that this deconvolution algorithm accounts for primary cortical neurons, thus allowing its usage in detecting neuronal cell death across neurodegenerative diseases.

## Brain-Derived cfDNA in Neurodegeneration

The application of cfDNA methylation-based analysis to neurodegenerative pathologies are still in their infancy. Research has consisted mostly of target-based studies, meaning that the detection of circulating brain-derived DNA relies on precisely measuring small regions, with specifically differential methylation behavior in the CNS in comparison to other tissues, mainly hematopoietic cells. A summary of the current data on CNS-derived cfDNA in a neurodegenerative context is presented in Table 1. The first such study that we are aware of is one by Lehmann-Werman et al. (2016), in which the authors first identified a pattern of clear demethylation in two clusters of CpGs in sorted oligodendrocytes’ methylome (located at the *MBP3* gene and in an unannotated region). By bisulfite sequencing such regions, they described increased oligodendrocyte-derived cfDNA in serum or plasma of multiple sclerosis patients. Using the same strategy (known unmethylation of a cluster of nine CpGs of brain tissue), they observed an increase in brain-derived cfDNA in cardiac arrest patients with documented brain damage, and also in traumatic brain injury patients (Lehmann-Werman et al., 2016). In line with this, Olsen et al. (2016b) demonstrated increased demethylation of the *MOG* gene in sera of active multiple sclerosis patients. In amyotrophic lateral sclerosis patients, plasma cfDNA methylation levels of the *RHBDF* promoter were increased, relative to healthy controls (Mendioroz et al., 2018). Lastly, Alzheimer’s patients have been shown to have higher levels of *LHX2* methylation in plasma-extracted cfDNA (Pai et al., 2019).

**Table 1:** Evidence of CNS-derived cfDNA in neurodegenerative settings.

**Condition**	**Cell or tissue of origin**	**DNA methylation pattern**	**Approach**	**Methodology**	**Reference**
**MS**	Oligodendrocyte	Demethylation at *MBP3* and unannotated region (CG10809560, *Illumina annotation*)	Target-specific	Bisulfite sequencing	Lehmann-Werman et al. (2016)
**Cardiac arrest with brain damage**	Brain	Demethylation at unannotated region (CG09787504, *Illumina annotation*)	Target-specific	Bisulfite sequencing	Lehmann-Werman et al. (2016)
**TBI**	Brain	Demethylation at unannotated region (CG09787504, *Illumina annotation*)	Target-specific	Bisulfite sequencing	Lehmann-Werman et al. (2016)
**MS**	Oligodendrocyte	Demethylation at *MOG*	Target-specific	Methylation-specific PCR	Olsen et al. (2016b)
**ALS**	Brain	Increased methylation at *RHBDF* promoter	Target-specific	Bisulfite sequencing	Mendioroz et al. (2018)
**AD**	Brain	Increased methylation at *LHX2*	Target-specific	Bisulfite sequencing	Pai et al. (2019)
**Explosive course trainees**	Neuron and glia	Differential DNA methylation in 33 regions	Multi-targeted	Bisulfite amplicon sequencing	Chatterton et al. (2021)

*AD, Alzheimer’s disease; ALS, Amyotrophic lateral sclerosis; MS, Multiple Sclerosis; TBI, Traumatic Brain Injury*.

In a more technically evolved and encompassing study, Chatterton et al. (2021) achieved an interesting proof-of-concept in which they were able to detect neuron and glia-derived cfDNA in human plasma samples. They developed a bisulfite amplicon sequencing protocol of a total of 33 regions with neuron and glial-specific DNA methylation patterns identified with an in-house pipeline from 450k microarray profiles. In blood plasma samples of entry personnel during a training course using explosives, the authors report evidence of neuron and glial cfDNA. Furthermore, significantly increased levels of neuron-derived cfDNA were observed on the day of training in which exposure to higher pressures was experienced (Chatterton et al., 2021).

Tissue biopsies are extremely invasive and, in the case of neurologic conditions, particularly inaccessible. Cerebrospinal fluid (CSF) analysis is an option but still causes major discomfort to patients. Along the neurodegeneration spectrum, there are multiple profiles of neurotrauma and damage. One can infer that CNS-derived cfDNA shed into the bloodstream may become a more accessible and interpretable tool for neurodegenerative biomarker research.

## Potential of cfDNA Methylome Profiling in Epilepsy

To the best of our knowledge, to date, there are only two studies on cfDNA studies in epilepsy. Both analyzing the concentration of cfDNA. Liimatainen et al. (2013) observed increased sera cfDNA concentration in 167 focal epilepsy patients vs. 250 healthy controls. Alapirtti et al. (2016) on the other hand, failed to verify differences between 52 refractory epilepsy patients (both focal and generalized) and 250 controls. One of the limitations of these studies is the heterogeneity of the studied populations, which include a large spectrum of epileptic syndromes. Given the variability within epilepsies and the increasingly evident differing etiopathogenic fingerprints between syndromes, more narrowly specified epilepsy cohorts would be of more relevance.

As for cfDNA methylation, nothing has, to the best of our knowledge, been reported yet. Nonetheless, altered DNA methylation patterns in brain tissue of epilepsy patients have been consistently described (Miller-Delaney et al., 2012; Liu et al., 2016; Wang et al., 2016; Zhang et al., 2021; Martins-Ferreira et al., 2022). Major efforts have been made for the use of DNA methylation as peripheral biomarkers, namely by demonstrating the correlation of DNA methylation patterns in brain tissue and peripheral tissues, such as blood, saliva, and buccal mucosa (Braun et al., 2019). Reinforcing the aforementioned idea, cfDNA methylation may be more representative of a specific pathological phenotype due to the link between cfDNA methylome, differing of tissue/cell contribution, and pattern of cell death, as described throughout this review.

Mesial Temporal Lobe Epilepsy (MTLE) is one of the most studied epilepsies due to its high incidence (it is the most common focal epilepsy in adults) and its high refractoriness rates. Hippocampal sclerosis is the most prevalent histopathological feature in MTLE. It is characterized by an exacerbated state of gliosis and a severe neuronal death landscape in the mesial temporal regions (Blümcke et al., 2013). A pertinent question regarding MTLE’s etiopathology is the time frame of HS occurrence. It has been considered a consequence of recurrent exposure to seizure activity. However, it can also be speculated that HS may have a role in promoting pro-epileptogenic activity onset and progression. This is, in part, due to the fact that the diagnosis often occurs in the chronic stage, upon the appearance of spontaneous recurrent seizures. The most recently reviewed definition of epileptogenesis describes it as a continuous event. After the initial precipitating injury, the brain undergoes a latent phase in which the ability to produce spontaneous recurrent seizures is acquired. The chronic stage, initiated upon the first unprovoked seizure, is no longer considered stationary, as was previously the case. It is rather a progressive phenomenon, with pathological molecular and cellular alterations extending as the disease progresses (Pitkänen et al., 2016). A recent study by our group has demonstrated major DNA methylation alterations in both hippocampus and anterior temporal neocortex of MTLE-HS patients in comparison to autopsied non-epileptic controls, with enrichment for a wide spectrum of epileptogenesis-related pathways. Additionally, we demonstrate a potential progressive remodeling of DNA methylation in inflammatory genes with increased disease duration (Martins-Ferreira et al., 2022).

**Figure 2:**
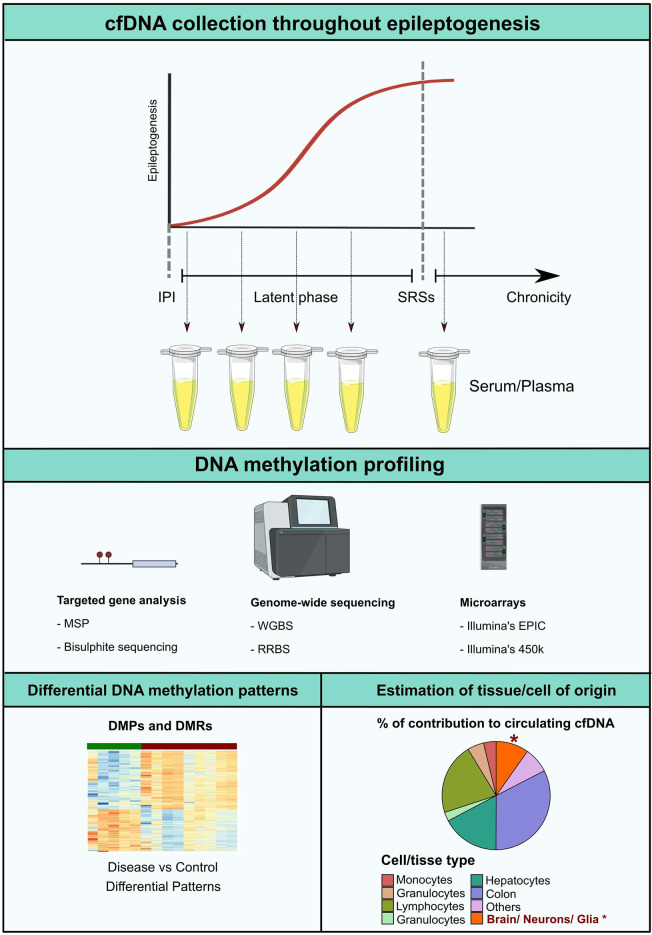
Epileptogenesis is considered to follow a progressive model. After the initial precipitating injury (IPI), the brain parenchyma enters a latent phase in which the ability to generate spontaneous recurrent seizures (SRSs) is established. In the chronic stage, upon initiation of unprovoked seizure activity, epileptogenic molecular and structural remodeling progress with time. DNA methylation profiling along the different timeframes of epileptogenesis would potentially represent a valuable approach for the development of novel non-invasive biomarkers. Possible analytical strategies include detection of altered cfDNA methylation patterns in pathological settings in comparison to controls, or estimation of the percentage of tissue or cell type contribution to the circulating cfDNA pool, which potentially insights on occurring CNS-specific cell death in the early stages of the disease. MSP, Methylation-Specific PCR; RRBS, reduced representation bisulfite sequencing; WBGS, whole genome bisulfite sequencing.

The main argument of interest of this review focus on the fact that cell death occurs in the MTLE-HS brain. Despite the time-point at which it may be occurring. Thus, it is plausible that neuron- or glia-derived cfDNA may be released into the circulation during neuro damage. We call for novel cfDNA methylation screening studies in epileptic patients, from targeted approaches like bisulfite sequencing and methylation-specific PCR to genome-wide screenings using microarrays, whole-genome bisulfite sequencing, and reduced representation bisulfite sequencing. Such studies would be potentially helpful in all stages of the epileptogenic landscape. It could promote detection of patients at high risk of epileptogenesis after a brain trauma incident and during latency, facilitate earlier diagnosis and better monitoring, and provide for therapeutic outcome prediction (Figure 2).

## Concluding Remarks

The research on circulating cfDNA has evolved exponentially during the last decades, from the proven clinical utility of genetic characterization to the emerging applications of DNA methylation. Identification of biomarkers is an urgent need across various pathologies, particularly in neurologic diseases in which tissue biopsies are extremely difficult to obtain or simply inaccessible. We propose that cfDNA methylation should be investigated in epileptic patients, as we consider it to be and intriguing and promising beam of light in what has been the gloomy field of epilepsy biomarker research.

## Author Contributions

All authors were responsible for the development of the conceptual map. RM-F performed literature search and drafted the manuscript. BL and PC were responsible for the critical revision and final approval of the final version to be published. All authors contributed to the article and approved the submitted version.

## Conflict of Interest

The authors declare that the research was conducted in the absence of any commercial or financial relationships that could be construed as a potential conflict of interest.

## Publisher’s Note

All claims expressed in this article are solely those of the authors and do not necessarily represent those of their affiliated organizations, or those of the publisher, the editors and the reviewers. Any product that may be evaluated in this article, or claim that may be made by its manufacturer, is not guaranteed or endorsed by the publisher.

## Abbreviations

cfDNA: cell-free DNA
DMP: differentially methylated position
DMR: differentially methylated region
HS: hippocampal sclerosis
MTLE: mesial temporal lobe epilepsy.
